# Health Tract, No. 178

**Published:** 1865

**Authors:** 


HEALTH TRACT, No. 178.

### WEATHER SIGNS.

SUDDEN changes of weather are the immediate cause of the sickness and death of multitudes, hence all persons owe it to themselves to study to some extent the portents of the heavens, from their own observation, as to the localities in which they live, paying but little attention, and relying not at all, on the signs of the weather as read in books, or detailed by others. Rules for farming and weather signs are proverbially uncertain and conflicting, arising from the one cause of applying observations of one locality to those of another. Many of the dates for planting in Minnesota would not answer in Louisiana. There are, however, some general signs which are applicable to all lands. Parents should begin early to draw the attention of their children to the weather signs of their individual localities ; this habit of observation will be largely valuable in other directions, in practical life.

The following lines are attributed to Dr. Jenner, written on declining an invitation to an excursion; these signs can be readily explained on strictly scientific principles:

"The hollow winds begin to blow,
The clouds look black, the glass is low,
The soot falls down, the spaniels sleep,
And spiders from their cobwebs creep.
Last night the sun went pale to bed,
The moon in halos hid her head ;
The boding shepherd heaves a sigh,
For see ! a rainbow spans the sky.
The walls are damp, the ditches smell,
Closed is the pink-eyed pimpernel.
Hark ! how the chairs and tables crack ;
Old Betty's joints are on the rack ;
Her corns with shooting pains torment her,
And to her bed untimely send her.
The smoke from chimneys right ascends,
Then spreading back to earth it bends.
The wind unsteady veers around,
Or settling in the south is found.
The tender colts on back do lie,
Nor heed the traveler passing by.
In fiery red the sun doth rise,
Then wades through clouds to mount the skies.
Loud quack the ducks, the peacocks cry,
The distant hills are looking nigh.
How restless are the snoring swine !
The busy flies disturb the kine.
Low o'er the grass the swallow wings ;
The cricket, too, how loud it sings ;
Puss, on the hearth, with velvet paws,
Sits smoothing o'er her whiskered jaws.
Through the clear stream the fishes rise,
And nimbly catch the incautious flies.
The sheep were seen, at early light,
Cropping the meads with eager bite.
Though June, the air is cold and chill ;
The mellow blackbird's voice is still ;
The glow-worms numerous and bright,
Illumed the dewy dell last night.
At dusk the squalid toad was seen,
Hopping, crawling o'er the green.
The frog has lost his yellow vest,
And in a dingy suit is dressed.
The leech, disturbed is newly risen,
Quite to the summit of his prison.
The whirling wind the dust obeys,
And in the rapid eddy plays.
My dog, so altered in his taste,
Quits mutton-bones, on grass to feast.
And see yon rooks ! how odd their flight !
They imitate the gliding kite ;
Or seem precipitate to fall,
As if they felt the piercing ball.
'Twill surely rain. I see with sorrow,
Our jaunt must be put off to-morrow."

